# NURSING HOME INVESTMENT IN HEALTH INFORMATION TECHNOLOGY – AN ORGANIZATIONAL SLACK PERSPECTIVE

**DOI:** 10.1093/geroni/igad104.3498

**Published:** 2023-12-21

**Authors:** John McHugh, Gregory Alexander

**Affiliations:** Columbia University, New York City, New York, United States; Columbia University, New York City, New York, United States

## Abstract

Organizational slack refers to excess resources (e.g., financial, social capital, human capital) an organization accumulates to adapt to changing internal and external contexts. Nursing homes operate in a severely resource-constrained environment, and therefore have limited ability to accumulate organizational slack. However, nursing homes that do accumulate slack may be more likely to invest in health information technology (HIT). To test this, we examine associations between organizational slack and HIT maturity. HIT maturity is the level of IT capabilities, extent of use, and integration in resident care, clinical support, and administration. We linked 2018 data from a national nursing home HIT maturity survey (N=525) to nursing home characteristics from LTCfocus.org, CMS data, National Center for Health Statistics, Medicare cost reports, and other county-level statistics. We incorporated Simple Linear Regression, controlling for facility-level sex, race, case-mix, and urban/rural location. Average 3-year operating income for Stage 0 nursing homes was -$4,387 per bed (n=7) compared to $42,768 per bed in Stage 6 nursing homes (n=17). Higher HIT maturity was associated with greater percentages of Medicare patients and shorter distances to the closest hospital. Our regression model also demonstrated significant associations with operating income per bed, chain affiliation, total beds, occupancy percentage, moderately concentrated counties and case-mix adjusted staffing. In this sample, larger, more financially stable organizations (i.e., those with more slack) may be able to invest more in HIT. As HIT maturity has been shown to be associated with improved patient outcomes, disparities may widen in organizations with lower levels of slack.

